# Investigating Variations in Medicine Approvals for Attention-Deficit/Hyperactivity Disorder: A Cross-Country Document Analysis Comparing Drug Labeling

**DOI:** 10.1177/10870547231224088

**Published:** 2024-02-07

**Authors:** Laila Tanana, Asam Latif, Prasad S Nishtala, Timothy F Chen

**Affiliations:** 1 School of Pharmacy, Faculty of Medicine and Health, The University of Sydney, Sydney, Australia; 2 University of Nottingham, Nottingham, UK; 3 Department of Life Sciences, University of Bath, Bath, UK

**Keywords:** ADHD, children, methylphenidate, psychostimulant, medication

## Abstract

**Objective:**

This study aimed to compare the approval of medicines for attention deficit/hyperactivity disorder (ADHD) for pediatric patients across five countries.

**Method:**

A document analysis was completed, using the drug labeling for ADHD medicines from five countries; United Kingdom, Australia, New Zealand, Canada and United States (US). Comparisons of available formulations and approval information for ADHD medicine use in pediatric patients were made.

**Results:**

The US had the highest number of approved medicines and medicine forms across the studied countries (29 medicine forms for 10 approved medicines). Approved age and dosage variations across countries and missing dosage information were identified in several drug labeling.

**Conclusions:**

The discrepancies in approval information in ADHD medicine drug labeling and differing availability of medicine formulations across countries suggest variations in the management of ADHD across countries. The update of drug labeling and further research into reasons for variability and impact on practice are needed.

## Introduction

Attention-deficit/hyperactivity disorder (ADHD) is a common childhood psychiatric disorder with an estimated pooled prevalence of 7.1% (Thomas et al., 2015). ADHD is characterized by impaired attention, motor hyperactivity and impulsivity, and symptoms often persist into adulthood which may impair social, academic and occupational functioning (Sibley et al., 2017; Thapar & Cooper, 2016). Diagnosis and subsequent prescribing and use of medicines to manage this condition in pediatric patients are increasing (Raman et al., 2018). However, the prevalence of ADHD medication use varies across countries and regions, appearing higher in North America (pooled prevalence of 4.48%) than in Northern Europe (1.95% pooled prevalence), Asia (0.95% pooled prevalence), Australia (0.95% pooled prevalence), and Western Europe (0.7% pooled prevalence) (Raman et al., 2018).

Where pharmacological treatment is indicated for ADHD, the medicine prescribed by a clinician may depend on where a person lives, local treatment guideline recommendations, and the availability and approval of medicines (Raman et al., 2018). Evidence suggests that there are variations in availability and ADHD medication use across different countries and regions, indicating differences in clinical approaches when managing ADHD (Okumura et al., 2019; Raman et al., 2018). For example, methylphenidate tends to be predominantly used in Australia, Canada, Asia, and European countries, whereas in the United States (US), the prescribing of amfetamines is more common or nearly as common as methylphenidate (Bachmann et al., 2017; Okumura et al., 2019; Raman et al., 2018; Song, 2016). In addition, nonstimulant medicines such as atomoxetine have been increasing in Korea and European countries but decreasing in the US (Bachmann et al., 2017; Song, 2016).

In most instances, the first-line pharmacological treatments recommended for ADHD are psychostimulant medicines, including methylphenidate, dexamfetamine, or amfetamines while nonstimulant medicines, such as atomoxetine, are recommended as second-line treatments (Bolea-Alamañac et al., 2014; Canadian ADHD Resource Alliance, 2020; National Institute for Health and Care Excellence, 2018; Pliszka, 2007; Taylor et al., 2018; Wolraich et al., 2019; Wolraich & Hagan, 2019). Third-line treatments include clonidine, imipramine, atypical antipsychotic, and other medicines (Canadian ADHD Resource Alliance, 2020). In the US, clonidine is recommended as a second-line pharmacological treatment, and it is also approved and marketed for ADHD (Wolraich & Hagan, 2019). In contrast, in Canada, Australia, and the United Kingdom (UK), it is not regulatory approved for use in pediatric ADHD, despite common use in practice (Ben Amor et al., 2014; Betts et al., 2014; Efron et al., 2013, 2017).

It is also important to note that the availability of different medication formulations is important for individualized treatment. However, the numerous formulations of psychostimulants available have led to confusion and errors during the prescribing and dispensing of these medicines (Chavez et al., 2009). This may also be of concern where patients may seek medicines when overseas or where prescribers may move practice across countries.

The labeling of a medicine (drug labeling) is authorized by regulatory authorities such as the Food and Drug Administration (FDA) in the US or the Therapeutics Goods Administration (TGA) in Australia. Health professionals may refer to the drug labeling during practice as they are a source of important clinical trial information and safety and dosage information. However, the drug labeling may differ across countries according to the requirements of different regulatory authorities. As the drug labeling and regulatory-approval of a medicine are supported by evidence of the safety and efficacy, it would be expected that the drug labeling would be standardized and consistent across countries. However, this does not always seem to be the case. For example, concerns have been raised about the differences in the publicly-available medication information for clonidine, where variations in regulatory-approved information could undermine patient adherence (Beckmann, 2013).

Little is known about the differences in approval of other ADHD medicines across countries, including more commonly used psychostimulants such as methylphenidate, dexamfetamine and lisdexamfetamine. An understanding of the use and availability of medicines in different countries may help contribute to the optimization of the management of ADHD in pediatric patients.

In light of the increasing prevalence of ADHD medication use and a lack of international comparisons of medicine approvals and formulations, this study aimed to compare the approval of ADHD medicines for pediatric patients across five countries; the UK, Australia, New Zealand (NZ), Canada, and the US.

## Methods

The ADHD medicines included in this study comprised two Anatomical Therapeutic Chemical (ATC) classification groups, namely “Psychoanaleptics (N06)” and “Antihypertensives (C02)” (World Health Organization, 2021).

The regulatory-approved drug labeling documents outline the approval of medicines in a country. These are referred to as the “Summary of Product Characteristics” in the UK, “Product Information” in Australia, “Data Sheet” in NZ, “Product Monograph” in Canada and “Prescribing Information” in the US. These documents were sourced from online databases (DataPharm Ltd., n.d.; Health Canada, n.d.; MEDSAFE New Zealand Medicines and Medical Devices Safety Authority, n.d.; Therapeutic Goods Administration, n.d.; U.S. National Library of Medicine, n.d.). It is recognized that several online databases contain drug labeling. The online databases used for this analysis are often used by healthcare professionals as they are easily accessible and provide efficient search methods for many medicines and their drug labeling.

Drug labeling were retrieved for each country in March 2023 and saved locally for data extraction and analysis; A summary of the references for the approved ADHD medicines and their drug labeling is available in Supplemental Table S1. The pediatric age group was defined as children and adolescents aged <18 years. Any differences in the spelling of medicine names across countries were overcome by standardization according to ATC code. Specifically, “amfetamine” is known as “amphetamine” and “dexamfetamine” is known as “dextroamphetamine” in the US and Canada, and metamfetamine is known as “methamphetamine” in the US.

**Figure 1.**
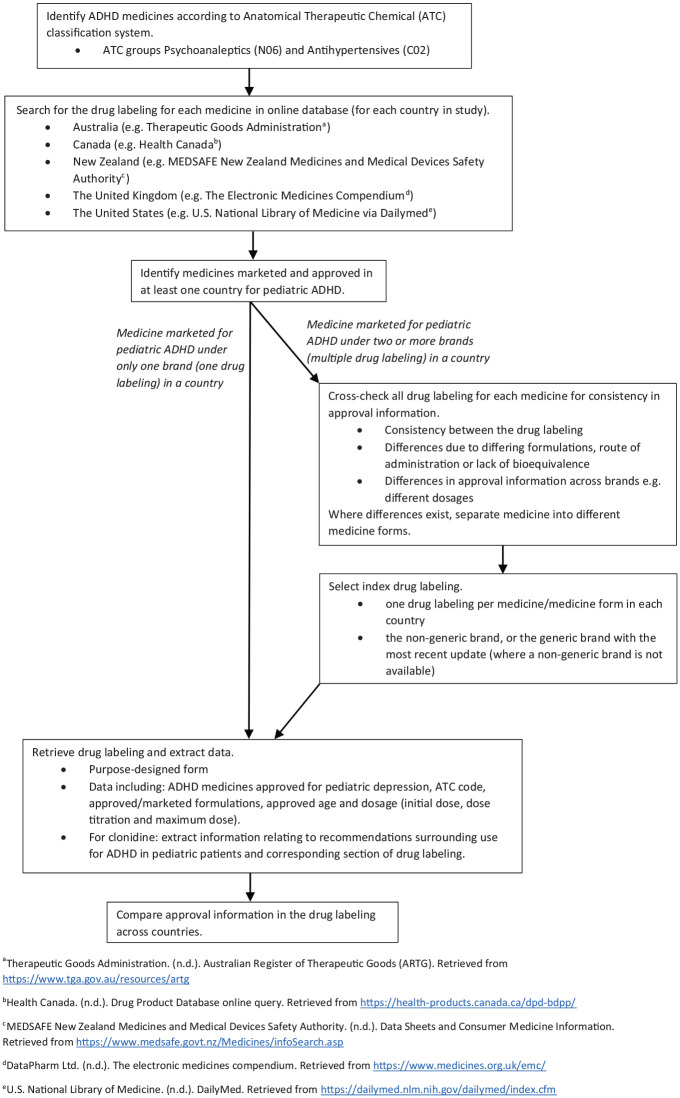
Process of identifying medicines for attention-deficit/hyperactivity disorder (ADHD) and drug labeling for cross-country comparisons of approval information. ^a^Therapeutic Goods Administration (n.d.). ^b^Health Canada (n.d.). ^c^ MEDSAFE New Zealand Medicines and Medical Devices Safety Authority (n.d.) ^d^DataPharm Ltd. (n.d.). ^e^U.S. National Library of Medicine. (n.d.).

The countries in our study were conveniently selected because they share similarities in Western culture, are high-income, are English-speaking, and have readily accessible regulatory documents online, in English. Except for the US, the other countries in our study (Australia, NZ, Canada, and the UK) also have largely public, national healthcare systems. In addition, given the strong evidence-based culture across these countries, one would expect there to be consistency in medicine guidelines and dosing. There is also significant immigration between these countries; hence, harmonizing regulatory documents and providing consistent health information is important. To address the aim of this study, a cross-comparison document analysis of drug labeling from five conveniently selected high-income countries was performed (Figure 1). All ADHD medicines comprising ATC groups N06 and C02 were searched in each online database and drug labeling were retrieved for the medicines approved for ADHD in pediatrics. Comparisons of all the approval information for pediatric ADHD were made across countries.

Where a medicine was marketed or approved in a country under more than one formulation or more than one trade name, resulting in multiple drug labeling, all documents were checked for consistency. Multiple preparations with variable immediate-release (IR), extended-release (XR) or delayed-release (DR) ratios were observed, particularly for methylphenidate and amfetamine. Formulation types included the following: capsule, chewable tablet, extended-release capsule (XR-C), extended-release chewable tablet, extended-release orally-disintegrating tablet (XR-ODT), extended-release suspension (XR-S), extended-release tablet (XR-T), solution, tablet and transdermal patch. Medicines and their formulations were separated into different medicine forms for analysis. During data analysis, only one drug labeling was assigned per medicine form per country; the non-generic brand or a generic brand with the most recent update (where a non-generic brand was unavailable).

Medicines with the same formulations were treated as equivalent when compared across countries, regardless of brand. Bioequivalence between Dyanavel^®^ XR-S and XR-T (*Dyanavel XR*, 2022), Evekeo^®^ and Evekeo ODT^®^ (*Evekeo*, 2022; *Evekeo ODT*, 2022), Procentra^®^ and Zenzedi^®^ (*Procentra*, 2022; *Zenzedi*, 2022), Vyvanse^®^ capsule and chewable tab (*Vyvanse*, 2020, 2022), Ritalin^®^ tab, Methylin^®^ solution and Methylphenidate IR chewable tab (*Methylin*, 2021; *Methylphenidate Hydrochloride*, 2021; *Ritalin/Ritalin-SR*, 2022) and Strattera^®^ capsule and solution (*Strattera*, 2021; *Strattera 4mg/mL oral solution*, 2021) were expected based on the information in the respective drug labeling. Quillichew ER^®^ (30% IR, 70% XR components) differs from Methylphenidate XR (CD) (Teva Pharmaceuticals US brand) (30% IR, 70% XR beads components) due to the pharmacokinetic profile and extended-release technology, so they were treated as different medicine forms (Childress et al., 2019; *Quillichew ER*, 2021). Methylphenidate XR (CD) (Teva Pharmaceuticals US brand) and Equasym XL^®^ (UK) showed similarity in their pharmacokinetics according to the drug labeling, so they were treated as the same medicine form in this study (*Equasym XL*, 2022; *Methylphenidate Hydrochloride (CD)*, 2022). Ritalin LA^®^ uses the SODAS^®^ (spheroidal oral drug absorption system) technology, while Medikinet^®^ XL uses modified-release capsules and both contain 50% IR and 50% XR components (*Medikinet XL*, 2021; *Ritalin/Ritalin-SR*, 2022). Medikinet^®^ XL has been shown to be bioequivalent to Ritalin LA^®^ under fasting conditions, but NOT in the fed state (Haessler et al., 2008). Dosages for Medikinet^®^ XL and Ritalin LA^®^ were compared under the same formulation for the purposes of this study.

Medicines with varying extended-release formulations were separated into different medicine forms based on IR to XR component ratios or XR technology as detailed in the drug labeling and/or the literature. Initially, the drug labeling was checked. Then, information was extracted from the literature where the drug labeling did not provide enough detail. Specifically, information from the literature was required to determine the IR/XR component ratios for Adderall XR^®^ (Greenhill et al., 2003; Swanson, 2005) and Mydayis^®^ (Takeda Pharmaceuticals U.S.A, 2023). Dyanavel^®^ XR (suspension and tablets) contain amfetamine IR and XR components, however, the ratios have not been described (Childress et al., 2019).

Data from the drug labeling were extracted using a purpose-designed form. They included the following information: medicine name, ATC code, approved/marketed formulations, approved age and approved dosage information (initial dose, dose titration and maximum dose). In addition, information relating to the use of clonidine in pediatric ADHD was also extracted, along with the corresponding section/subsection of the drug labeling. Data extraction and analysis were performed by one author (L.T.), and 10% of the data set was independently checked for accuracy by another author (A.L.). Where discrepancies existed, a discussion was held until a consensus was reached and adjudicated by a third reviewer (T.C.).

## Results

Ten medicines from ATC groups N06 and C02 were approved for pediatric ADHD in one or more of the studied countries (amfetamine, atomoxetine, clonidine, dexamfetamine, dexmethylphenidate, dexmethylphenidate and serdexmethylphenidate, guanfacine, lisdexamfetamine, metamfetamine, and methylphenidate) (Table 1). From these medicines, 29 different medicine forms were identified and included in the analysis (including immediate-release and extended-release formulations, capsules, chewable tablets, orally-disintegrating tablets, solutions, suspensions, and transdermal patches), comprising a total of 62 drug labeling across the studied countries. All 29 medicine forms were approved in the US, with 11 approvals in Canada, 8 in the UK, 7 in Australia, and 7 in NZ (Table 2).

**Table 1.** Approval of Medicines for ADHD in Pediatric Patients Across Five Countries.

Country	Australia	Canada	New Zealand	United Kingdom	United States
Total number of medicine approvals	5	6	4	5	10
Approved centrally acting sympathomimetics (ATC N06BA)	Atomoxetine, dexamfetamine, lisdexamfetamine, methylphenidate	Amfetamine, atomoxetine, dexamfetamine, lisdexamfetamine, methylphenidate	Atomoxetine, dexamfetamine, lisdexamfetamine methylphenidate	Atomoxetine, dexamfetamine, lisdexamfetamine, methylphenidate	Amfetamine, atomoxetine, dexamfetamine, dexmethylphenidate, dexmethylphenidate and serdexmethylphenidate lisdexamfetamine, metamfetamine, methylphenidate
Approved imidazoline receptor agonists (ATC C02AC)	Guanfacine	Guanfacine	*None*	Guanfacine	Clonidine, guanfacine

**Table 2.** Medicine Forms Approved for ADHD in Pediatric Patients Across Five Countries.

Medicine and ATC code	Medicine form	Approved formulations and trade names in each country				
		Australia	Canada	New Zealand	United Kingdom	United States
Amfetamine (N06BA01)	Amfetamine IR					tablet (Evekeo^®^ ) ≥3 years ODT (Evekeo ODT^®^ ) ≥6 years
	Amfetamine XR (50% IR, 50% DR)					XR-ODT (Adzenys XR-ODT^™^ ) ≥6 years
	Amfetamine XR (IR/XR component ratio unknown)^a^					XR-S, XR-T (Dyanavel^®^ XR) ≥6 years
	Amfetamine mixed salts IR					tablet (Adderall^®^ ) ≥3 years
	Amfetamine mixed salts XR (50% IR, 50 % DR)		XR-C (Adderall XR^®^ ) ≥6 years			XR-C (Adderall XR^®^ ) ≥6 years
	Amfetamine mixed salts XR (IR/DR/DR; equal ratios)^b^					XR-C (Mydayis^®^ ) ≥13 years
Dexamfetamine (N06BA02)	Dexamfetamine IR	tablet (Aspen Dexamfetamine) ≥3 years	tablet (Dexedrine^®^ ) ≥6 years	tablet (Dexamfetamine) ≥3 years	tablet (Amfexa^®^ ), solution ≥6 years	tablet (Zenzedi^®^ ), solution (Procentra^®^ ) ≥3 years
	Dexamfetamine XR (sustained release formulation)		XR-C (Dexedrine^®^ Spansule^®^ ) ≥6 years			XR-C (Dexedrine^®^ Spansule^®^ ) ≥6 years
	Dexamfetamine transdermal patch					Transdermal patch (Xelstrym) ≥6 years
Metamfetamine (N06BA03)	Metamfetamine IR					tablet (Desoxyn^®^ ) ≥6 years
Methylphenidate (N06BA04)	Methylphenidate transdermal patch					Transdermal patch (Daytrana^®^ ) ≥6 years
	Methylphenidate IR	tablet (Ritalin^®^ ) ≥6 years	tablet (pms-methylphenidate) ≥6 years	tablet (Ritalin^®^ ) ≥6 years	tablet (Medikinet^®^ ) ≥6 years	tablet (Ritalin^®^ ), solution (Methylin^®^ ), chew tab (LUPIN generic brand) ≥6 years
	Methylphenidate XR (sustained release formulation)		XR-T (APO-methylphenidate SR) ≥6 years	XR-T (Rubifen^®^ SR) ≥6 years		XR-T (Ritalin-SR^®^ ) ≥6 years
	Methylphenidate XR (outer DR coating/inner XR coating)^c^					XR-C (Jornay PM^®^ ) ≥6 years
	Methylphenidate XR (20% IR, 80% XR components)					XR-S (Quillivant XR^®^ ) ≥6 years
	Methylphenidate XR (multilayer beads: 20% IR layer, 80% XR layer)		XR-C (Foquest^®^ ) ≥6 years			XR-C (Adhansia XR^®^ ) ≥6 years
	Methylphenidate XR (22% IR, 78% XR components)	XR-T (Concerta^®^ ) ≥6 years	XR-T (Concerta^®^ ) ≥6 years	XR-T (Concerta^®^ ) ≥6 years	XR-T (Concerta^®^ XL) ≥6 years	XR-T (Concerta^®^ ) ≥6 years
	Methylphenidate XR (25% IR, 75% XR components)					XR-ODT (Cotempla XR-ODT^™^ ) ≥6 years
	Methylphenidate XR (30% IR, 70% XR components)					XR-chew tab (Quillichew ER^®^ )^d^ ≥6 years
	Methylphenidate XR (30% IR, 70% XR bead components)				XR-C (Equasym XL) ≥6 years	XR-C (CD TEVA generic brand)^d^ 6-15 years
	Methylphenidate XR (40% IR, 60% XR)		XR-C (Biphentin^®^ ) ≥6 years			XR-C (Aptensio XR^®^ ) ≥6 years
	Methylphenidate XR (50% IR, 50% XR)^e^	XR-C (Ritalin LA^®^ ) ≥6 years		XR-C (Ritalin LA^®^ ) ≥6 years	XR-C (Medikinet^®^ XL)^e^ ≥6 years	XR-C (Ritalin LA^®^ ) ≥6 years
Atomoxetine (N06BA09)	Atomoxetine IR	capsule (Strattera^®^ ) ≥6 years	capsule (Strattera^®^ ) ≥6 years	capsule (Strattera^®^ ) ≥6 years	capsule (Strattera^®^ ), solution (Strattera^®^ solution) ≥6 years	capsule (Strattera^®^ ) ≥6 years
Dexmethylphenidate (N06BA11)	Dexmethylphenidate IR					tablet (Focalin^®^ ) ≥6 years
	Dexmethylphenidate XR (50% IR, 50% DR)					XR-C (Focalin XR^®^ ) ≥6 years
Lisdexamfetamine (N06BA12)	Lisdexamfetamine IR	capsule (Vyvanse^®^ ) ≥6 years	capsule (Vyvanse^®^ ), chew tab (Vyvanse^®^ ) ≥6 years	capsule (Vyvanse^®^ ) ≥6 years	capsule (Elvanse^®^ ) ≥6 years	capsule (Vyvanse^®^ ), chew tab (Vyvanse^®^ ) ≥6 years
Dexmethylphenidate and Serdexmethylphenidate (N06BA15)	Dexmethylphenidate and Serdexmethylphenidate IR					capsule (Azstarys^™^ ) ≥6 years
Clonidine (C02AC01)	Clonidine XR (extended release formulation)					XR-T (Kapvay^®^ ) ≥6 years
Guanfacine (C02AC02)	Guanfacine XR (extended release formulation)	XR-T (Intuniv^®^ ) ≥6 years	XR-T (Intuniv XR^®^ ) ≥6 years		XR-T (Intuniv^®^ ) ≥6 years	XR-T (Intuniv^®^ ) ≥6 years

*Note*. Gray fill: Not marketed in the respective country. chew tab = chewable tablet; XR = extended-release; XR-C = extended-release capsule; XR-chew tab = extended-release chewable tablet; XR-ODT = extended-release orally-disintegrating tablet; XR-S = extended-release suspension; XR-T = extended-release tablet; IR = immediate-release; ODT = orally-disintegrating tablet.

a Dyanavel XR^®^ (solution and tablets) contain both IR and XR components of amfetamine, although the ratio of IR to XR is unknown. Reference: Childress et al. (2020).

b *“. . .They contain three types of drug-releasing beads, an immediate release and two different types of delayed release (DR) beads. The first DR bead releases amphetamine at pH 5.5 and the other DR bead releases amphetamine at pH 7.0.”* Reference: *Mydayis* (2022).

c *“. . .JORNAY PM extended-release capsules contain beads with two functional film coatings (outer delayed-release and inner extended-release) surrounding a drug core coated with methylphenidate hydrochloride. The outer, delayed-release coating delays the initial release of methylphenidate while the inner extended-release coating controls the release throughout the day. . .”, “. . .The initial absorption of methylphenidate into plasma is delayed such that no more than 5% of total drug is available within the first 10* *hours after dosing. After the lag period, the absorption of methylphenidate occurs in a single peak with a median T 14.0* *hours, followed by a gradual decline throughout the rest of the day. . .”* Reference: *Jornay PM extended-release* (2022).

d Quillichew ER^®^ (30% IR, 70% XR components) differs from methylphenidate CD TEVA brand (30% IR, 70% XR beads components) due to the pharmacokinetic profile and extended-release technology, so they were treated as different drug formulations.

e Medikinet^®^ LA has shown to be bioequivalent to Ritalin LA under fasting conditions, but NOT in the fed state. Reference: Haessler et al. (2008).

Five of the 62 drug labeling had the approval for the ADHD indication for children as young as 3 years. These included amfetamine IR (Evekeo^®^) and dexamfetamine IR (Procentra^®^ and Zenzedi^®^) in the US, dexamfetamine IR (Aspen dexamfetamine) in Australia and dexamfetamine IR in NZ (*Aspen Dexamfetamine*, 2021; *Dexamfetamine Tablets*, 2017; *Evekeo*, 2022; *Procentra*, 2022; *Zenzedi*, 2022). Amfetamine mixed salts XR (Mydayis^®^) in the US was approved for adolescents 13 years and older (*Mydayis*, 2022). Methylphenidate XR (CD) (Teva Pharmaceuticals US brand) was approved for children 6 to 15 years old (*Methylphenidate Hydrochloride (CD)*, 2022). All other medicines were approved for children and adolescents 6 years and older (across 55 drug labeling). Dexamfetamine was approved for use in all five countries but there were inconsistencies in the approved ages in the drug labeling; 3 years and older in Australia (*Aspen Dexamfetamine*, 2021), NZ (*Dexamfetamine Tablets*, 2017) and the US (*Focalin*, 2022), and 6 years and older in the UK (*Dexamfetamine Sulfate 1 mg/ mL Oral Solution*, 2022) and Canada (*Dexedrine/Dexedrine Spansule*, 2020). All other medicines approved in two or more countries were consistent in the approved ages.

The dosage information was missing or incomplete in eight drug labeling, most of which were from the US (Adderall XR^®^, Desoxyn^®^, Zenzedi^®^ and Procentra^®^), dexamfetamine in NZ, Ritalin^®^ in Australia and Strattera^®^ and Strattera^®^ oral solution in the UK (Table 3). The Canada drug labeling for Concerta^®^ provided the dose titration as “Adjust at weekly intervals.” While the specific doses were not mentioned, Concerta^®^ is available in 18 mg, 27 mg, 36 mg, 54 mg and 72 mg strengths, which we would expect to guide those dosage adjustments. Similarly, while the US drug labeling for Daytrana^®^did not specify a maximum dosage, the 30 mg/9 hour patches are the highest strength available, which we would expect to indicate the maximum dose.

**Table 3.** Missing Dosage Information in Drug Labeling for Pediatric ADHD Medicines.

Medicine form	Trade name	Country	Missing information in drug labeling	Drug labeling reference
Amfetamine mixed salts XR (50% IR, 50 % XR)	Adderall XR^®^	United States	Maximum dose not specified for ≥13 years (Maximum dose provided for 6–12 years).	*Adderall XR* (2022)
Metamfetamine hydrochloride IR	Desoxyn^®^	United States	Maximum dose not specified.	*Desoxyn* (2022)
Dexamfetamine IR	Dexamfetamine	New Zealand	Maximum dose not specified.	*Dexamfetamine Tablets* (2017)
Dexamfetamine IR	Procentra^®^	United States	Maximum dose not specified for 3–5 years of age (Maximum dose provided for ≥6 years).	*Procentra* (2022)
Methylphenidate IR	Ritalin^®^	Australia	Maximum dose not specified for immediate-release formulation (Ritalin^®^).	*Ritalin 10/Ritalin LA* (2022)
Atomoxetine IR	Strattera^®^	United Kingdom	Dose titration not specified for <70 kg bodyweight (Dose titration provided for >70 kg bodyweight).	*Strattera* (2021)
Atomoxetine IR	Strattera^®^ 4 mg/mL oral solution	United Kingdom	Dose titration not specified for <70 kg bodyweight (Dose titration provided for >70 kg bodyweight).	*Strattera 4mg/mL oral solution* (2021)
Dexamfetamine IR	Zenzedi^®^	United States	Maximum dose not specified for 3–5 years (Maximum dose provided for ≥6 years).	*Zenzedi* (2022)

*Note*. Full dosage information can be accessed in Supplemental Table 2. XR = extended-release; IR = immediate-release.

Five of 29 medicine forms were approved in all five countries; atomoxetine IR, dexamfetamine IR, lisdexamfetamine IR, Methylphenidate IR, and Methylphenidate XR (Concerta^®^/Concerta^®^ XL). Inconsistencies in the approved dosage information in the drug labeling exist across the studied countries (Table 4). A full description of the approved ages, initial dose, dose titration and maximum doses extracted from the drug labeling for all the approved ADHD medicines is available in Supplemental Table S2.

**Table 4.** Dosage Comparisons for ADHD Medicines Approved in All Five Countries (Australia, Canada, New Zealand, the United Kingdom, and the United States).

Medicine form	Australia	Canada	New Zealand	United Kingdom	United States
Atomoxetine IR	Strattera^®^ capsules Approved age: ≥6 years Initial dose: ≤70 kg bodyweight: 0.5 mg/kg daily, >70 kg bodyweight: 40 mg daily. Give daily doses as mane or b.d.; morning and late afternoon or early evening. Dose titration: After a minimum of 3 days, increase to a target dose of approximately 1.2 mg/kg/day (≤70 kg bodyweight) or 80 mg daily (>70 kg bodyweight). After 2–4 weeks, may increase the above to respective maximum dose. Maximum dose: ≤70 kg bodyweight: 1.4 mg/kg or 100 mg daily, whichever is less. >70 kg bodyweight: 100 mg daily.	Strattera^®^ capsules Approved age: ≥6 years Initial dose: ≤70 kg bodyweight: 0.5 mg/kg daily, >70 kg bodyweight: 40 mg daily. Give daily doses as mane or b.d.; morning and late afternoon or early evening. Dose titration: After 7–14 days, increase to 0.8 mg/kg/day (≤70 kg bodyweight) or 60 mg daily (>70 kg bodyweight). After another 7–14 days, increase to approximately 1.2 mg/kg/day (≤70 kg bodyweight) or 80 mg daily (>70 kg bodyweight). After a minimum of 30 days, reassess and adjust maintenance dose (≤70 kg bodyweight) or for patients >70 kg, may increase to maximum dose after 2–4 weeks if necessary. Maximum dose: ≤70 kg bodyweight: 1.4 mg/kg or 100 mg daily, whichever is less. >70 kg bodyweight: 100 mg daily.	Strattera^®^ capsules Approved age: ≥6 years Initial dose: ≤70 kg bodyweight: 0.5 mg/kg daily, >70 kg bodyweight: 40 mg daily. Give daily doses as mane or b.d.; morning and late afternoon or early evening. Dose titration: After a minimum of 3 days, increase to a target dose of approximately 1.2 mg/kg/day (≤70 kg bodyweight) or 80 mg daily (>70 kg bodyweight). After 2–4 weeks, may increase the above to respective maximum dose. Maximum dose: ≤70 kg bodyweight: 1.4 mg/kg or 100 mg daily, whichever is less. >70 kg bodyweight: 100 mg daily.	Strattera^®^ capsules and oral solution Approved age: ≥6 years Initial dose: ≤70 kg bodyweight: 0.5 mg/kg daily, >70 kg bodyweight: 40 mg daily. Give daily doses as mane or b.d.; morning and late afternoon or early evening. Dose titration: After a minimum of 7 days, increase to a maintenance dose of approximately 1.2 mg/kg/day (≤70 kg bodyweight) or 80 mg daily (>70 kg bodyweight). Maximum dose: ≤70 kg bodyweight: Dose not specified.^a^ For >70 kg bodyweight: 100 mg daily.	Strattera^®^ capsules Approved age: ≥6 years Initial dose: ≤70 kg bodyweight: 0.5 mg/kg daily, >70 kg bodyweight: 40 mg daily. Give daily doses as mane or b.d.; morning and late afternoon or early evening. Dose titration: After a minimum of 3 days, increase to a target dose of approximately 1.2 mg/kg/day (≤70 kg bodyweight) or 80 mg daily (>70 kg bodyweight). After 2–4 weeks, may increase to maximum dose (>70 kg bodyweight). Maximum dose: ≤70 kg bodyweight: 1.4 mg/kg or 100 mg daily, whichever is less. >70 kg bodyweight: 100 mg daily.
Dexamfetamine IR	Aspen dexamfetamine tablets Approved age: ≥3 years Initial dose: 2.5 mg daily. Dose titration: 2.5 mg daily at weekly intervals. Maximum dose: 40 mg daily in two divided doses.	Dexedrine^®^ tablets Approved age: ≥6 years Initial dose: 5 mg o.d. or b.d. Dose titration: 5 mg daily at weekly intervals. Maximum dose: Only in rare cases will it be necessary to exceed a total of 40 mg per day.	Dexamfetamine tablets Approved age: ≥3 years Initial dose: 3–5 years: 2.5 mg daily, ≥6 years: 5 mg o.d. or b.d. Dose titration: 3–5 years: 2.5 mg daily at weekly intervals, ≥6 years: 5 mg daily at weekly intervals. Maximum dose: Dose not specified.^a^	Amfexa^®^ tablets, dexamfetamine oral solution Approved age: ≥6 years Initial dose: 5 mg o.d. or b.d. Dose titration: 5 mg daily at weekly intervals. Maximum dose: Usually 20 mg daily, although 40 mg daily may be needed in rare cases.	Zenzedi^®^ tablets, Procentra^®^ oral solution Approved age: ≥3 years Initial dose: 3–5 years: 2.5 mg daily, ≥6 years: 5 mg o.d. or b.d. Dose titration: 3–5 years: 2.5 mg daily at weekly intervals, ≥6 years: 5 mg daily at weekly intervals. Maximum dose: 3–5 years: Dose not specified.^a^ ≥6 years: Only in rare cases will it be necessary to exceed a total of 40 mg per day.
Lisdexamfetamine IR	Vyvanse^®^ capsules Approved age: ≥6 years Initial dose: 30 mg mane (or 20 mg if deemed appropriate) Dose titration: 20 mg daily at intervals no more frequently than weekly Maximum dose: 70 mg daily	Vyvanse^®^ capsules and chewable tablets Approved age: ≥6 years Initial dose: 30 mg mane (or 20 mg if deemed appropriate) Dose titration: 10 or 20 mg daily at approx. weekly intervals. Maximum dose: 60 mg daily	Vyvanse^®^ capsules Approved age: ≥6 years Initial dose: 30 mg mane Dose titration: 20 mg daily at intervals no more frequently than weekly Maximum dose: 70 mg daily	Elvanse^®^ capsules Approved age: ≥6 years Initial dose: 30 mg mane (or 20 mg if deemed appropriate) Dose titration: 10 or 20 mg daily at approx. weekly intervals. Maximum dose: 70 mg daily	Vyvanse^®^ capsules and chewable tablets Approved age: ≥6 years Initial dose: 30 mg mane Dose titration: 10 or 20 mg daily at approx. weekly intervals. Maximum dose: 70 mg daily
Methylphenidate IR	Ritalin^®^ tablets Approved age: ≥6 years Initial dose: 5 mg o.d. or b.d. Dose titration: 5–10 mg daily at weekly intervals Maximum dose: Dose not specified^a^	Pms-Methylphenidate tablets Approved age: ≥6 years Initial dose: 5 mg t.d.s. Dose titration: 5–10 mg daily at weekly intervals Maximum dose: 60 mg daily	Ritalin^®^ tablets Approved age: ≥6 years Initial dose: 5 mg o.d. or b.d. Dose titration: 5–10 mg daily at weekly intervals Maximum dose: 60 mg daily	Medikinet^®^ tablets Approved age: ≥6 years Initial dose: 5 mg o.d. or b.d. Dose titration: 5–10 mg daily at weekly intervals Maximum dose: 60 mg daily	Ritalin^®^ tablets, Methylin^®^ oral solution, Methylphenidate chewable tablets (generic brand) Approved age: ≥6 years Initial dose: 5 mg b.d. Dose titration: 5–10 mg daily at weekly intervals Maximum dose: 60 mg daily
Methylphenidate XR (22% IR, 78% XR components)	Concerta^®^ XR-tablets Approved age: ≥6 years Initial dose: new to methylphenidate: 18 mg mane. Switching from IR methylphenidate: 18 mg (previously 5 mg t.d.s. IR) or 36 mg (previously 10 mg t.d.s. IR) or 54 mg (previously 15 mg t.d.s. IR) mane. Dose titration: 9 mg daily (from 18–36 mg) at weekly intervals, and then 18 mg daily at weekly intervals. Maximum dose: ≥6 years: 54 mg mane.	Concerta^®^ XR-tablets Approved age: ≥6 years Initial dose: new to methylphenidate: 18 mg mane. switching from IR methylphenidate: 18 mg (previously 5 mg b.d.-t.d.s. IR or 20 mg SR daily) or 36 mg (previously 10 mg b.d.-t.d.s. IR or 40 mg SR daily) or 54 mg (previously 15 mg b.d.-t.d.s. IR or 60 mg SR daily). Dose titration: Adjust at weekly intervals.^e^ Maximum dose: ≥6 years: 54 mg mane for patients new to methylphenidate. Dose not specified^a^ for patients switching from a current methylphenidate regimen.	Concerta^®^ XR-tablets Approved age: ≥6 years Initial dose: new to methylphenidate: 18 mg mane. Switching from IR methylphenidate: 18 mg (previously 5 mg t.d.s. IR) or 36 mg (previously 10 mg t.d.s. IR) or 54 mg (previously 15 mg t.d.s. IR) or 72 mg (previously 20 mg t.d.s. IR) mane. Dose titration: 9 mg daily (from 18 to 36 mg) at weekly intervals, and then 18 mg daily at weekly intervals. Maximum dose: 6–12 years: 54 mg mane, ≥13 years: 72 mg mane.	Concerta^®^ XL XR-tablets Approved age: ≥6 years Initial dose: new to methylphenidate: 18 mg mane. Switching from IR methylphenidate: 18 mg (previously 5 mg t.d.s. IR) or 36 mg (previously 10 mg t.d.s. IR) or 54 mg (previously 15 mg t.d.s. IR) mane. Dose titration: 18 mg daily at weekly intervals. Maximum dose: ≥6 years: 54 mg mane.	Concerta^®^ XR-tablets Approved age: ≥6 years Initial dose: new to methylphenidate: 18 mg mane. switching from IR methylphenidate: 18 mg (previously 5 mg b.d.-t.d.s IR) or 36 mg (previously 10 mg b.d.-t.d.s IR) or 54 mg (previously 15 mg b.d.-t.d.s IR) or 72 mg (previously 20 mg b.d.-t.d.s IR) mane. Dose titration: 18 mg daily at weekly intervals. Maximum dose: 6–12 years: 54 mg mane, ≥13 years: 72 mg mane, not to exceed 2 mg/kg/day.

a Dose not specified in corresponding drug labeling.

*Note*. XR = extended-releasel; XR-tablets = extended-release tablets; IR = immediate-release; o.d. = once daily; mane = once daily in the morning; t.d.s. = three times daily; b.d. = twice daily.

Clonidine oral formulations include immediate-release and extended-release preparations across the studied countries and of these, only clonidine XR (Kapvay^®^) is approved for pediatric ADHD in the US (Table 2). Excerpts from the drug labeling relating to the use of clonidine for pediatric ADHD identified inconsistencies in the drug labeling between the US (clonidine XR) and other countries (clonidine IR in the UK, Australia, NZ, and Canada) (Table 5). The US drug labeling advises that the safety and efficacy of clonidine for use in pediatric ADHD have been established in pediatric patients. In contrast, the drug labeling in other countries (UK, Australia, NZ, and Canada) signify a lack of safety and efficacy in this population. It is important to note that the formulation approved in the US (XR) differs from that of other countries (IR) for pediatric ADHD. Furthermore, the US drug labeling for clonidine IR does not include information on its use in pediatric ADHD (*Clonidine Hydrochloride*, 2023).

**Table 5.** Excerpts From Drug Labeling of Clonidine Relating to Use in Pediatric ADHD.

Clonidine immediate-release oral formulation			
Country and drug labeling	Drug labeling excerpt	Location of excerpt in drug labeling	Drug labeling reference
Australia	“The use and the safety of clonidine in children and adolescents has little supporting evidence in randomised controlled trials and therefore cannot be recommended for use in this population. In particular, when clonidine is used off-label concomitantly with methylphenidate in children with ADHD, serious adverse reactions, including death, have been observed. Therefore, clonidine in this combination is not recommended.”	4. CLINICAL PARTICULARS (Subsection: 4.4 Special warnings and precautions for use)	*Catapres* (2021)
Canada	“*[methylphenidate]* The concomitant use with clonidine has resulted in serious adverse reactions, including death, in children with attention-deficit/hyperactivity (ADHD).”	DRUG INTERACTIONS	*Teva-Clonidine* (2014)
New Zealand	“The use and the safety of clonidine in children and adolescents has little supporting evidence in randomised controlled trials and therefore cannot be recommended for use in this population. In particular, when clonidine is used off-label concomitantly with methylphenidate in children with ADHS, serious adverse reactions, including death, have been observed. Therefore, clonidine in this combination is not recommended.”	4. CLINICAL PARTICULARS (Subsection: 4.4 Special warnings and precautions for use)	*Catapres* (2020)
United Kingdom	“The efficacy of clonidine has also been investigated in a few clinical studies with paediatric patients with ADHD, Tourette syndrome and stuttering. The efficacy of clonidine in these conditions has not been demonstrated.”	5. PHARMACOLOGICAL PROPERTIES (Subsection: 5.1 pharmacodynamic properties)	*Clonidine 25mcg Tablets BP* (2020). *Clonidine Hydrochloride 50mcg/5mL Oral Solution* (2020).
United States	*None*	*N/A*	*Clonidine Hydrochloride* (2023)
Clonidine extended-release oral formulation^a,b^			
Country	Drug labeling excerpt	Location of excerpt in drug labeling	Drug labeling reference
United States^b^	“The safety and efficacy of KAPVAY in the treatment of ADHD have been established in pediatric patients 6 to 17 years of age. Use of KAPVAY in pediatric patients 6 to 17 years of age is supported by three adequate and well-controlled studies; a short-term, placebo-controlled monotherapy trial, a short-term adjunctive therapy trial and a longer-term randomized monotherapy trial *[see Clinical Studies (14)].* Safety and efficacy in pediatric patients below the age of 6 years has not been established.”	8 USE IN SPECIFIC POPULATIONS (Subsection: 8.4 Pediatric Use)	*Kapvay* (2022)
	“Efficacy of KAPVAY in the treatment of ADHD was established in children and adolescents (6 to 17 years) in: • One short-term, placebo-controlled monotherapy trial (Study 1) • One short-term adjunctive therapy to psychostimulants trial (Study 2) • One randomized withdrawal trial as monotherapy (Study 3). . .”	14 CLINICAL STUDIES	

a Clonidine XR oral formulation is only marketed in the United States, thus drug labeling were not available for this formulation in Australia, Canada, New Zealand and the United Kingdom.

b Additional information relating to the approval of clonidine extended-release oral tablet for pediatric ADHD can be found in the United States drug labeling.

## Discussion

Our study provides a five-country cross-comparison of the approval of ADHD medicines for pediatric patients. Our findings indicate differences in the approval and availability of ADHD medicines and their formulations across countries and conflicting information about clonidine for use in pediatric ADHD in the drug labeling. A lack of dosage information was identified in several drug labeling. Our findings identify a need for the review and update of the relevant information in the drug labeling of ADHD medicines.

We found differences in the number of approved ADHD medicines and available formulations across the studied countries. In particular, we found the highest number of approved ADHD medicines and formulations were available in the US (29 medicine forms available for 10 medicines), where pediatric ADHD diagnoses are highest (9.4%) (Danielson et al., 2018). In the US, prescribing guidelines recommend the use of methylphenidate (preschool children) or FDA-approved psychostimulants (aged 6 years and older) as first-line pharmacological agents, which is supported by the availability of a range of appropriate formulations, such as oral suspensions, orally-disintegrating tablets, chewable tablets or transdermal patches (Subcommittee on Attention-Deficit/Hyperactivity Disorder Steering Committee on Quality Improvement and Management, 2011; Wolraich et al., 2019; Wolraich & Hagan, 2019). Australia, Canada, NZ, and the UK have a much smaller population than the US, which could explain the fewer formulations and medicines available. Except for an oral solution for atomoxetine (*Strattera 4* *mg/mL oral solution*, 2021) and dexamfetamine (*Dexamfetamine Sulfate 1* *mg/ mL Oral Solution*, 2022) in the UK and lisdexamfetamine chewable tablets in Canada (*Vyvanse*, 2020), we found that immediate-release and extended-release tablet and capsule formulations for ADHD medicines dominate in the UK, Australia, NZ, and Canada, despite the approval and need for use in young children as recommended in prescribing guidelines (Canadian ADHD Resource Alliance, 2020; National Institute for Health and Care Excellence, 2018; Taylor et al., 2018). Concerns have been raised about a lack of availability and accessibility to appropriate formulations of psychostimulants for young children (Schirm et al., 2003). In addition, dosing inconvenience, swallowing difficulties and social stigma contribute to ADHD treatment discontinuation in pediatric patients (Gajria et al., 2014). Although the drug labeling recommend that some capsules can be opened and dispersed into food or drinks, this carries a risk of unreliable dosing or incomplete dose delivery (Childress et al., 2020). Where alternative formulations which are not commercially available may be required, for example, oral suspensions or dispersible formulations for children with swallowing difficulties (Cortese et al., 2017; Gajria et al., 2014), special pharmaceutical compounding may be required, however, this may not be easily accessible and is often substantially more expensive for patients.

The differences in approval of ADHD medicines and formulations across countries identified in our study could also result in the need for prescribers in other countries to change a patient’s therapy should they travel or move overseas. This could be confusing or distressing for the patient and their family, especially where unnecessary changes to treatment are made. As the population and prevalence of pediatric ADHD differs across countries, and the market size changes, this probably influences the number of marketed products and available formulations. Prescribers and patients/carers will need to work together to manage changes in medicines, should they patient move to a country with different medicine and formulation availabilities. The findings of this study may be helpful to improve clinicians’ awareness of these issues, particularly for those who may be moving their practice overseas, or managing patients who have arrived from abroad.

Amfetamines were approved in children as young as 3 years old in our study; amfetamine IR (Evekeo^®^ in the US), amfetamine mixed salts IR (Adderall^®^ in the US) and dexamfetamine in Australia, NZ, and the US. Interestingly, methylphenidate has more extensive research supporting its safe and efficacious use in the 3 to 5-year age group (Kollins et al., 2006). However, methylphenidate, along with the other ADHD medicines identified in our study, were approved by regulatory agencies for children 6 years and older in the studied countries (Supplemental Table S2). While the regulatory-approved age in the drug labeling may influence a prescriber’s decision, off-label prescribing may be deemed necessary in practice, and if so, it should align with current evidence and sufficient experience using the medicines (General Medical Council, 2019; Sugarman et al., 2013). Symptoms of ADHD are, however, often difficult to distinguish from highly variable normative behaviors before the age of 4 years (American Psychiatric Association, 2022) and treatment is more prevalent in pediatric patients 6 years and over, which corresponds with the larger number of ADHD medicine approvals for this age group identified in our study. In the US, 2% of children 2 to 5 years are estimated to be diagnosed with ADHD, and 18% receive ADHD medicines. Comparatively, 10% children 6 to 11 years are diagnosed with ADHD, and 69% of them receive ADHD medicines, while 13% of adolescents aged 13 to 17 are diagnosed with ADHD, and 62% of them receive ADHD medicines (Centres for Disease Control and Prevention, 2022).

In line with findings from other studies, we encountered missing or incomplete dosage information (particularly maximum dosage and dose titration guidelines) in several drug labeling (Arguello et al., 2014; Beers et al., 2013; Salgado et al., 2013; San Miguel et al., 2005; Weersink et al., 2019). Our analysis found that the maximum dosage was absent in six drug labeling and the dose titration guidelines were incomplete in another two drug labeling (Table 3). The maximum doses in drug labeling can provide a guide to healthcare professionals during the prescribing and supply of medicines. A recent meta-analysis found a range of recommended maximum doses for methylphenidate used in clinical studies but no discernible scientific justification for any recommendations (Ching et al., 2019). There are also concerns that prescribers may not adhere to regulatory or prescribing guidelines in practice (Epstein et al., 2014). Further, deficiencies in the content information in drug labeling may contribute to variability in practice and elicit uncertainty among healthcare professionals utilizing the drug labeling in practice, with concerns about the reliability of the included information. Therefore, there is a need to review and update the content information in drug labeling of ADHD medicines to ensure the completeness of the included information.

Interestingly, we identified differences in the guidelines for dose titration in the drug labeling of Concerta^®^/Concerta^®^ XL (Methylphenidate XR) across the five approved countries (the UK, Australia, NZ, Canada, and the US), despite marketing by the same pharmaceutical company, Janssen. Similarly, the dosage information for Strattera^®^ (atomoxetine) differs across the drug labeling from the approved countries, despite being marketed by Eli Lilly and Company in all the countries. Previous studies have also identified differences in the content information of drug labeling for ADHD medicines (Aagaard & Hansen, 2013; Warrer et al., 2014) and antidepressants across countries (Tanana et al., 2021). Our findings raise questions about how such discrepancies can materialize and could potentially indicate variations in the prescribing and management of ADHD across countries. Regulatory processes may vary across countries, which could explain the differences in the content information in the drug labeling. Further research may be needed to determine why differences in drug labeling exist across countries and any impacts on practice.

Clonidine (extended-release) is approved for ADHD in pediatric patients either as monotherapy or an adjunct to stimulants in the US. On the other hand, other clonidine (immediate-release) drug labeling comment on lacking evidence to support the efficacy of clonidine in ADHD (Australia, NZ, and the UK drug labeling) and provide a warning that concomitant use of clonidine with methylphenidate is associated with serious adverse events, including death, so the combination is not recommended (Australia, Canada, and NZ drug labeling) (Table 5). Comparatively, the US drug labeling for clonidine (immediate-release) does not comment on its use in ADHD (*Clonidine Hydrochloride*, 2023). Despite only being approved in the US for pediatric ADHD, clonidine is commonly prescribed to pediatric ADHD patients in the US, Canada, and Australia (Ben Amor et al., 2014; Betts et al., 2014; Efron et al., 2013). Guidelines recommend the use of clonidine for ADHD in certain circumstances, as a third-line medicine (Canadian ADHD Resource Alliance, 2020; Taylor et al., 2018), as a second-line agent (Wolraich & Hagan, 2019) or with advice from a tertiary ADHD service (National Institute for Health and Care Excellence, 2018). Furthermore, studies have determined the efficacy and well-tolerated use of clonidine for ADHD in pediatric patients (Catalá-López et al., 2017; Childress et al., 2020; Health Products Regulatory Authority, n.d.; Ming et al., 2011). Drug labeling are publicly available online, meaning that patients and carers from different countries can access them. Such conflicting information in the drug labeling and outdated safety information could undermine medication adherence (Beckmann, 2013). Changes to the current drug labeling to reflect updated safety information and improved consistency in the included approval information across countries may be warranted.

Regulatory agencies have implemented changes in recent years to improve the format of drug labeling and to harmonize drug labeling with international regulators (New Zealand Medicines and Medical Devices Safety Authority, 2017; Therapeutic Goods Administration, 2020). Our international comparisons identify other areas for consideration during the update of drug labeling and support the decisions of regulatory bodies wishing to improve global harmonization of regulatory documents, such as the Australian TGA (Therapeutic Goods Administration, 2020) and New Zealand Medicines and Medical Devices Safety Authority (New Zealand Medicines and Medical Devices Safety Authority, 2017). Furthermore, the international standardization of medicine names used in drug labeling could help to overcome potential confusion in practice, especially when using prescribing guidelines from different countries. For example, “dexamfetamine” is the medicine name used in Australia, NZ, and the UK, but “dextroamphetamine” is used in the US and Canada. Our study concept also aligns with the aims of The International Council for Harmonization of Technical Requirements for Pharmaceuticals for Human Use (ICH), to improve regulatory process harmonization worldwide (The International Council for Harmonisation, n.d.).

Our study had some limitations. Our findings are limited to the five countries in our analysis, so they may not be generalizable to other developed, low or middle-income countries. It is acknowledged that drug labeling change over time, necessitating an update to these findings in the future. We recognize that only one researcher extracted all of the data, so a second author randomly and independently checked 10% of the data set to provide robustness. No significant discrepancies between the two authors were noted. Additionally, we acknowledge that medicines marketed for each formulation did not necessarily indicate bioequivalence between countries. However, we utilized research in the current literature and a detailed analysis of the drug labeling to justify brand comparisons across countries.

## Conclusion

Overall, our study identified missing dosage information and variability in the approval information in drug labeling for some ADHD medicines across countries. Additionally, we noted differences in the number of approved ADHD medicines and their marketed formulations across countries, which could indicate variations in the management of ADHD across countries and implications for those who may want to travel or live in another country. We also identified a need for the update of the drug labeling to reflect current evidence about safety and efficacy (i.e., for clonidine use in ADHD). Prescribers and patients require reliable and consistent information to inform them of the appropriate selection of a medicine, its formulation and appropriate dosage for the management of ADHD in pediatric patients. The similarities in Western culture between the five English-speaking countries selected in our study, as well as the similarities in their public national healthcare systems (except that of the US) may lead us to expect uniformity in the regulatory approval of medicines. However, we identified differences in approved medicines, age groups and regulatory information, which raise questions about the reasons for variability in drug labeling, prompting the need for further research. Considering the vulnerable nature of the pediatric age group, an improvement in the consistency of drug labeling across these Western countries could hopefully encourage the harmonization of regulatory information across other Western and non-Western countries. Better harmonization of regulatory information may reduce variability in the management of pediatric ADHD across different countries, thereby optimizing patient care and treatment outcomes.

## Supplemental Material

sj-docx-1-jad-10.1177_10870547231224088 – Supplemental material for Investigating Variations in Medicine Approvals for Attention-Deficit/Hyperactivity Disorder: A Cross-Country Document Analysis Comparing Drug Labeling Supplemental material, sj-docx-1-jad-10.1177_10870547231224088 for Investigating Variations in Medicine Approvals for Attention-Deficit/Hyperactivity Disorder: A Cross-Country Document Analysis Comparing Drug Labeling by Laila Tanana, Asam Latif, Prasad S Nishtala and Timothy F Chen in Journal of Attention Disorders

sj-docx-2-jad-10.1177_10870547231224088 – Supplemental material for Investigating Variations in Medicine Approvals for Attention-Deficit/Hyperactivity Disorder: A Cross-Country Document Analysis Comparing Drug Labeling Supplemental material, sj-docx-2-jad-10.1177_10870547231224088 for Investigating Variations in Medicine Approvals for Attention-Deficit/Hyperactivity Disorder: A Cross-Country Document Analysis Comparing Drug Labeling by Laila Tanana, Asam Latif, Prasad S Nishtala and Timothy F Chen in Journal of Attention Disorders
