# Supplementary material for: Investigating Variations in Medicine Approvals for Attention-Deficit/Hyperactivity Disorder: A Cross-Country Document Analysis Comparing Drug Labeling
Source: J Atten Disord. 2024 Feb 7;28(11):1437–53. doi: 10.1177/10870547231224088 (PMC11328451; doi:10.1177/10870547231224088)
Supplement: sj-docx-1-jad-10.1177_10870547231224088 – Supplemental material for Investigating Variations in Medicine Approvals for Attention-Deficit/Hyperactivity Disorder: A Cross-Country Document Analysis Comparing Drug Labeling [file sj-docx-1-jad-10.1177_10870547231224088.docx]

**Supplemental Table S1**

A summary of the reference information for the drug labelling of ADHD medicines approved for use in paediatric patients

| **Medicine and ATC code** | **Medicine form** | **Australia** | **Canada** | **New Zealand** | **United Kingdom** | **United States** |
| --- | --- | --- | --- | --- | --- | --- |
| Amfetamine (ATC N06BA01) | Amfetamine IR |  |  |  |  | *Tablet*  *Evekeo* (2022). Prescribing Information. Arbor Pharmaceuticals. Retrieved March 8, 2023, from <https://dailymed.nlm.nih.gov/dailymed/index.cfm>  *ODT*  *Evekeo ODT* (2022). Prescribing Information. Azurity Pharmaceuticals, Inc. Retrieved March 8, 2023, from <https://dailymed.nlm.nih.gov/dailymed/index.cfm> |
|  | Amfetamine XR (50% IR, 50% DR) |  |  |  |  | *XR-ODT*  *Adzenys XR-ODT* (2022). Prescribing Information. Neos Therapeutics Brands, LLC. Retrieved March 8, 2023, from <https://dailymed.nlm.nih.gov/dailymed/index.cfm> |
|  | Amfetamine XR (IR/XR component ratio unknown) |  |  |  |  | *XR-S, XR-T*  *Dyanavel XR* (2022). Prescribing Information. Nextwave Pharmaceuticals, Inc. Retrieved March 8, 2023, from <https://dailymed.nlm.nih.gov/dailymed/index.cfm> |
|  | Amfetamine mixed salts IR |  |  |  |  | *Tablet*  *Adderall* (2022). Prescribing Information. Teva Pharmaceuticals USA, Inc. Retrieved March 8, 2023, from <https://dailymed.nlm.nih.gov/dailymed/index.cfm> |
|  | Amfetamine mixed salts XR (50% IR, 50 % DR) |  | *XR-C*  *Adderall XR* (2022). Product Monograph. Takeda Canada Inc. Retrieved March 8, 2023, from <https://health-products.canada.ca/dpd-bdpp/> |  |  | *XR-C*  *Adderall XR* (2022). Prescribing Information. Takeda Pharmaceuticals America, Inc. Retrieved March 8, 2023, from <https://dailymed.nlm.nih.gov/dailymed/index.cfm> |
|  | Amfetamine mixed salts XR (IR/DR/DR; equal ratios) |  |  |  |  | *XR-C*  *Mydayis* (2022). Prescribing Information. Takeda Pharmaceuticals America, Inc. Retrieved March 8, 2023, from <https://dailymed.nlm.nih.gov/dailymed/index.cfm> |
| Dexamfetamine (ATC N06BA02) | Dexamfetamine IR | *Tablet*  *Aspen Dexamfetamine* (2021). Product Information. Aspen Pharma Pty Ltd. Retrieved March 8, 2023, from <https://www.tga.gov.au/resources/artg> | *Tablet*  *Dexedrine/Dexedrine Spansule* (2020). Product Monograph. Paladin Labs Inc. Retrieved March 8, 2023, from <https://health-products.canada.ca/dpd-bdpp/> | *Tablet*  *Dexamfetamine Tablets* (2017). New Zealand Data Sheet. PSM Healthcare Limited, t/a API Consumer Brands. Retrieved March 8, 2023, from <https://www.medsafe.govt.nz/Medicines/infoSearch.asp> | *Tablet*  *Amfexa* (2022). Summary of Product Characteristics. Medice UK LTD. Retrieved March 8, 2023, from <https://www.medicines.org.uk/emc/>  *Oral solution*  *Dexamfetamine Sulfate 1 mg/ ml Oral Solution* (2022). Summary of Product Characteristics. Rosemont Pharmaceuticals Limited. Retrieved March 8, 2023, from <https://www.medicines.org.uk/emc/> | *Tablet*  *Zenzedi* (2022). Prescribing Information. Arbor Pharmaceuticals, Inc. Retrieved March 8, 2023, from <https://dailymed.nlm.nih.gov/dailymed/index.cfm>  *Oral solution*  *Procentra* (2022). Prescribing Information. Independence Pharmaceuticals, LLC. Retrieved March 8, 2023, from <https://dailymed.nlm.nih.gov/dailymed/index.cfm> |
|  | Dexamfetamine XR (sustained release formulation) |  | *XR-C*  *Dexedrine/Dexedrine Spansule* (2020). Product Monograph. Paladin Labs Inc. Retrieved March 8, 2023, from <https://health-products.canada.ca/dpd-bdpp/> |  |  | *XR-C*  *Dexedrine Spansule* (2022). Prescribing Information. Amneal Pharmaceuticals LLC. Retrieved March 8, 2023, from <https://dailymed.nlm.nih.gov/dailymed/index.cfm> |
|  | Dexamfetamine transdermal patch |  |  |  |  | *Transdermal patch*  *Xelstrym* (2023). Prescribing Information. Noven Therapeutics, LLC. Retrieved March 8, 2023, from <https://dailymed.nlm.nih.gov/dailymed/index.cfm> |
| Metamfetamine (ATC N06BA03) | Metamfetamine IR |  |  |  |  | *Tablet*  *Desoxyn* (2022). Prescribing Information. Key Therapeutics. Retrieved March 8, 2023, from <https://dailymed.nlm.nih.gov/dailymed/index.cfm> |
| Methylphenidate (ATC N06BA04) | Methylphenidate transdermal patch |  |  |  |  | *Transdermal patch*  *Daytrana* (2022). Prescribing Information. Noven Therapeutics, LLC. Retrieved March 8, 2023, from <https://dailymed.nlm.nih.gov/dailymed/index.cfm> |
|  | Methylphenidate IR | *Tablet*  *Ritalin 10/Ritalin LA* (2022). Product Information. Novartis Pharmaceuticals Australia Pty Ltd. Retrieved March 8, 2023, from <https://www.tga.gov.au/resources/artg> | *Tablet*  *pms-Methylphenidate* (2022). Product Monograph. Pharmascience Inc. Retrieved March 28, 2023, from <https://health-products.canada.ca/dpd-bdpp/> | *Tablet*  *Ritalin/Ritalin LA* (2022). New Zealand Data Sheet. Novartis New Zealand Limited. Retrieved March 8, 2023, from <https://www.medsafe.govt.nz/Medicines/infoSearch.asp> | *Tablet*  *Medikinet* (2022). Summary of Product Characteristics. Medice UK LTD. Retrieved March 8, 2023, from <https://www.medicines.org.uk/emc/> | *Tablet*  *Ritalin/Ritalin-SR* (2022). Prescribing Information. Novartis Pharmaceuticals Corporation. Retrieved March 8, 2023, from <https://dailymed.nlm.nih.gov/dailymed/index.cfm>  *Oral solution*  *Methylin* (2021). Prescribing Information. Shionogi Inc. Retrieved March 8, 2023, from <https://dailymed.nlm.nih.gov/dailymed/index.cfm>  *Chewable tablet*  *Methylphenidate Hydrochloride* (2021). Prescribing Information. Lupin Pharmaceutics, Inc. Retrieved March 28, 2023, from <https://dailymed.nlm.nih.gov/dailymed/index.cfm> |
|  | Methylphenidate XR (sustained release formulation) |  | *XR-T*  *APO-Methylphenidate/APO-Methylphenidate SR* (2020). Product Monograph. Apotex Inc. Retrieved March 28, 2023, from <https://health-products.canada.ca/dpd-bdpp/> | *XR-T*  *Rubifen/Rubifen SR* (2022). New Zealand Data Sheet. AFT Pharmaceuticals Limited. Retrieved March 8, 2023, from <https://www.medsafe.govt.nz/Medicines/infoSearch.asp> |  | *XR-T*  *Ritalin/Ritalin-SR* (2022). Prescribing Information. Novartis Pharmaceuticals Corporation. Retrieved March 8, 2023, from <https://dailymed.nlm.nih.gov/dailymed/index.cfm> |
|  | Methylphenidate XR (outer DR coating/inner XR coating) |  |  |  |  | *XR-C*  *Jornay PM extended-release* (2022). Prescribing Information. Ironshore Pharmaceuticals Inc. Retrieved March 8, 2023, from <https://dailymed.nlm.nih.gov/dailymed/index.cfm> |
|  | Methylphenidate XR (20% IR, 80% XR components) |  |  |  |  | *XR-S*  *Quillivant XR* (2021). Prescribing Information. NextWave Pharmaceuticals, Inc. Retrieved March 8, 2023, from <https://dailymed.nlm.nih.gov/dailymed/index.cfm> |
|  | Methylphenidate XR (multilayer beads: 20% IR layer, 80% XR layer) |  | *XR-C*  *Foquest* (2022). Product Monograph. Elvium Life Sciences. Retrieved March 28, 2023, from <https://health-products.canada.ca/dpd-bdpp/> |  |  | *XR-C*  *Adhansia XR* (2022). Prescribing Information. Adlon Therapeutics L.P. Retrieved March 8, 2023, from <https://dailymed.nlm.nih.gov/dailymed/index.cfm> |
|  | Methylphenidate XR (22% IR, 78% XR components) | *XR-T*  *Concerta* (2021). Product Information. Janssen-Cilag Pty Ltd. Retrieved March 8, 2023, from <https://www.tga.gov.au/resources/artg> | *XR-T*  *Concerta* (2022). Product Monograph. Janssen Inc. Retrieved March 8, 2023, from <https://health-products.canada.ca/dpd-bdpp/> | *XR-T*  *Concerta* (2022). New Zealand Data Sheet. Janssen-Cilag (New Zealand) Ltd. Retrieved March 8, 2023, from <https://www.medsafe.govt.nz/Medicines/infoSearch.asp> | *XR-T*  *Concerta XL* (2022). Summary of Product Characteristics. Janssen-Cilag Ltd. Retrieved March 8, 2023, from <https://www.medicines.org.uk/emc/> | *XR-T*  *Concerta* (2023). Prescribing Information. Janssen Pharmaceuticals, Inc. Retrieved March 8, 2023, from <https://dailymed.nlm.nih.gov/dailymed/index.cfm> |
|  | Methylphenidate XR (25% IR, 75% XR components) |  |  |  |  | *XR-ODT*  *Cotempla XR-ODT* (2022). Prescribing Information. Neos Therapeutics Brands, LLC. Retrieved March 8, 2023, from <https://dailymed.nlm.nih.gov/dailymed/index.cfm> |
|  | Methylphenidate XR (30% IR, 70% XR components) |  |  |  |  | *XR-chewable tablet*  *Quillichew ER* (2021). Prescribing Information. NextWave Pharmaceuticals, Inc. Retrieved March 8, 2023, from <https://dailymed.nlm.nih.gov/dailymed/index.cfm> |
|  | Methylphenidate XR (30% IR, 70% XR bead components) |  |  |  | *XR-C*  *Equasym XL* (2022). Summary of Product Characteristics. Takeda UK Ltd. Retrieved March 8, 2023, from <https://www.medicines.org.uk/emc/> | *XR-C*  *Methylphenidate Hydrochloride (CD)* (2022). Prescribing Information. Teva Pharmaceuticals USA, Inc. Retrieved March 8, 2023, from <https://dailymed.nlm.nih.gov/dailymed/index.cfm> |
|  | Methylphenidate XR (40% IR, 60% XR) |  | *XR-C*  *Biphentin* (2021). Product Monograph. Elvium Life Sciences. Retrieved March 8, 2023, from <https://health-products.canada.ca/dpd-bdpp/> |  |  | *XR-C*  *Aptensio XR* (2023). Prescribing Information. Rhodes Pharmaceuticals L.P. Retrieved March 8, 2023, from <https://dailymed.nlm.nih.gov/dailymed/index.cfm> |
|  | Methylphenidate XR (50% IR, 50% XR) | *XR-C*  *Ritalin 10/Ritalin LA* (2022). Product Information. Novartis Pharmaceuticals Australia Pty Ltd. Retrieved March 8, 2023, from <https://www.tga.gov.au/resources/artg> |  | *XR-C*  *Ritalin/Ritalin LA* (2022). New Zealand Data Sheet. Novartis New Zealand Limited. Retrieved March 8, 2023, from <https://www.medsafe.govt.nz/Medicines/infoSearch.asp> | *XR-C*  *Medikinet XL* (2021). Summary of Product Characteristics. Medice UK LTD. Retrieved March 8, 2023, from <https://www.medicines.org.uk/emc/> | *XR-C*  *Ritalin LA* (2022). Prescribing Information. Novartis Pharmaceuticals Corporation. Retrieved March 8, 2023, from <https://dailymed.nlm.nih.gov/dailymed/index.cfm> |
| Atomoxetine (ATC N06BA09) | Atomoxetine IR | *Capsule*  *Strattera* (2020). Product Information. Eli Lilly Australia Pty Ltd. Retrieved March 8, 2023, from <https://www.tga.gov.au/resources/artg> | *Capsule*  *Strattera* (2015). Product Monograph. Eli Lilly Canada Inc. Retrieved March 8, 2023, from <https://health-products.canada.ca/dpd-bdpp/> | *Capsule*  *Strattera* (2020). New Zealand Data Sheet. Eli Lilly and Company (NZ) Limited. Retrieved March 8, 2023, from <https://www.medsafe.govt.nz/Medicines/infoSearch.asp> | *Capsule*  *Strattera* (2021). Summary of Product Characteristics. Eli Lilly and Company Limited. Retrieved March 8, 2023, from <https://www.medicines.org.uk/emc/>  *Oral solution*  *Strattera 4mg/mL oral solution* (2021). Summary of Product Characteristics. Eli Lilly and Company Limited. Retrieved March 8, 2023, from <https://www.medicines.org.uk/emc/> | *Capsule*  *Strattera* (2022). Prescribing Information. Eli Lilly and Company. Retrieved March 27, 2023, from <https://dailymed.nlm.nih.gov/dailymed/index.cfm> |
| Dexmethylphenidate (ATC N06BA11) | Dexmethylphenidate IR |  |  |  |  | *tablet*  *Focalin* (2022). Prescribing Information. Novartis Pharmaceuticals Corporation. Retrieved March 8, 2023, from <https://dailymed.nlm.nih.gov/dailymed/index.cfm> |
|  | Dexmethylphenidate XR (50% IR, 50% DR) |  |  |  |  | *XR-C*  *Focalin XR* (2022). Prescribing Information. Novartis Pharmaceuticals Corporation. Retrieved March 27, 2023, from <https://dailymed.nlm.nih.gov/dailymed/index.cfm> |
| Lisdexamfetamine (ATC N06BA12) | Lisdexamfetamine IR | *Capsule*  *Vyvanse* (2021). Product Information. Takeda Pharmaceuticals Australia Pty Ltd. Retrieved March 8, 2023, from <https://www.tga.gov.au/resources/artg> | *Capsule, chewable tablet*  *Vyvanse* (2020). Product Monograph. Takeda Canada Inc. Retrieved March 8, 2023, from <https://health-products.canada.ca/dpd-bdpp/> | *Capsule*  *Vyvanse* (2022). New Zealand Data Sheet. Takeda New Zealand Limited. Retrieved March 8, 2023, from <https://www.medsafe.govt.nz/Medicines/infoSearch.asp> | *Capsule*  Elvanse (2022). Summary of Product Characteristics. Takeda UK Ltd. Retrieved March 8, 2023, from <https://www.medicines.org.uk/emc/> | *Capsule, chewable tablet*  *Vyvanse* (2022). Prescribing Information. Takeda Pharmaceuticals America, Inc. Retrieved March 8, 2023, from <https://dailymed.nlm.nih.gov/dailymed/index.cfm> |
| Dexmethylphenidate and Serdexmethylphenidate  (ATC N06BA15) | Dexmethylphenidate and Serdexmethylphenidate IR |  |  |  |  | *Capsule*  *Azstarys* (2021). Prescribing Information. Corium, Inc. Retrieved March 8, 2023, from <https://dailymed.nlm.nih.gov/dailymed/index.cfm> |
| Clonidine (ATC C02AC01) | Clonidine XR (extended release formulation) |  |  |  |  | *XR-T*  *Kapvay* (2022). Prescribing Information. Concordia Pharmaceuticals Inc. Retrieved March 27, 2023, from <https://dailymed.nlm.nih.gov/dailymed/index.cfm> |
| Guanfacine (ATC C02AC02) | Guanfacine XR (extended release formulation) | *XR-T*  *Intuniv* (2021). Product Information. Takeda Pharmaceuticals Australia Pty Ltd. Retrieved March 8, 2023, from <https://www.tga.gov.au/resources/artg> | *XR-T*  *Intuniv XR* (2020). Product Monograph. Takeda Canada Inc. Retrieved March 8, 2023, from <https://health-products.canada.ca/dpd-bdpp/> |  | *XR-T*  *Intuniv* (2023). Summary of Product Characteristics. Takeda UK Ltd. Retrieved March 8, 2023, from <https://www.medicines.org.uk/emc/> | *XR-T*  *Intuniv* (2022). Prescribing Information. Takeda Pharmaceuticals America, Inc. Retrieved March 27, 2023, from <https://dailymed.nlm.nih.gov/dailymed/index.cfm> |

Abbreviations: immediate-release (IR), extended-release (XR), extended-release capsule (XR-C), extended-release orally disintegrating tablet (XR-ODT), extended-release suspension (XR-S), extended-release tablet (XR-T), orally-disintegration tablet (ODT)

Grey fill: Not marketed in respective country
