# Supplementary material for: Investigating Variations in Medicine Approvals for Attention-Deficit/Hyperactivity Disorder: A Cross-Country Document Analysis Comparing Drug Labeling
Source: J Atten Disord. 2024 Feb 7;28(11):1437–53. doi: 10.1177/10870547231224088 (PMC11328451; doi:10.1177/10870547231224088)
Supplement: sj-docx-2-jad-10.1177_10870547231224088 – Supplemental material for Investigating Variations in Medicine Approvals for Attention-Deficit/Hyperactivity Disorder: A Cross-Country Document Analysis Comparing Drug Labeling [file sj-docx-2-jad-10.1177_10870547231224088.docx]

**Supplemental Table S2**

Comparison of drug labeling approval information for pediatric ADHD medicines across five countries.

| **Medicine form** | **Australia** | **Canada** | **New Zealand** | **United Kingdom** | **United States** |
| --- | --- | --- | --- | --- | --- |
| Amfetamine IR |  |  |  |  | **Evekeo®**  Approved age: ≥3 years  Initial dose: *3-5 years:* 2.5 mg daily, *≥6 years:* 5 mg o.d. or b.d. (first dose on awakening and additional 1 or 2 doses at 4-6 hour intervals).  Dose titration: *3-5 years:* 2.5 mg daily at weekly intervals, *≥6 years:* 5 mg daily at weekly intervals.  Max dose: *3-5 years:* Dose not specified.^a^  *≥6 years:* Only in rare cases will it be necessary to exceed a total of 40 mg per day.  **Evekeo ODT®**  Approved age: ≥6 years  Initial dose: 5 mg o.d. or b.d.  Dose titration: 5 mg daily at weekly intervals.  Max dose: Only in rare cases will it be necessary to exceed a total of 40 mg per day. |
| Amfetamine XR (50% IR, 50% DR) |  |  |  |  | **Adzenys XR-ODT™**  Approved age: ≥6 years  Initial dose: 6.3 mg o.d., except when switching from Adderall XR; may continue at equivalent doses (3.1, 6.3, 9.4, 12.5, 15.7 or 18.8 mg if previously using 5, 10, 15, 20, 25 or 30 mg of Adderall XR, respectively).  Dose titration: 3.1mg or 6.3mg at weekly intervals  Max dose: *6-12 years:* 18.8 mg daily, *13-17 years:* 12.5 mg daily |
| Amfetamine XR (IR/XR component ratio unknown)^b^ |  |  |  |  | **Dyanavel® XR**  Approved age: ≥6 years  Initial dose: 2.5 or 5 mg o.d.  Dose titration: 2.5-10 mg daily at intervals of 4-7 days.  Max dose: 20 mg daily |
| Amfetamine mixed salts IR |  |  |  |  | **Adderall®**  Approved age: ≥3 years  Initial dose: *3-5 years:* 2.5 mg daily, *≥6 years:* 5 mg o.d. or b.d. (first dose on awakening and additional 1 or 2 doses at 4-6 hour intervals).  Dose titration: *3-5 years:* 2.5 mg daily at weekly intervals,  *≥6 years:* 5 mg daily at weekly intervals.  Max dose: *3-5 years:* Dose not specified.^a^  *≥6 years:* Only in rare cases will it be necessary to exceed a total of 40 mg per day. |
| Amfetamine mixed salts XR (50% IR, 50 % DR) |  | **Adderall XR®**  Approved age: ≥6 years  Initial dose: *6-12 years:* Usually 10 mg mane, or 5 mg if a lower dose is deemed appropriate, *≥13 years:* 10 mg mane  Dose titration: ≥*6 years:* 5-10 mg daily at weekly intervals.  Max dose: *6-12 years:* 30 mg daily. *≥13 years:* Usually 20 mg daily. In some cases, higher doses up to 30 mg daily may be required. |  |  | **Adderall XR®**  Approved age: ≥6 years  Initial dose: Usually 10 mg mane, or 5 mg if a lower dose is deemed appropriate, *≥13 years:* 10 mg mane.  Dose titration: *6-12 years:* 5-10 mg daily at weekly intervals. *≥13 years:* May increase to 20 mg mane after one week if necessary.  Max dose: *6-12 years:* 30 mg daily, *≥13 years:* Dose not specified.^a^ |
| Amfetamine mixed salts XR (IR/DR/DR; equal ratios)^c^ |  |  |  |  | **Mydayis®**  Approved age: ≥13 years  Initial dose: 12.5 mg mane  Dose titration: 12.5 mg daily at intervals no sooner than weekly.  Max dose: 25 mg daily |
| Metamfetamine IR |  |  |  |  | **Desoxyn®**  Approved age: ≥6 years  Initial dose: 5 mg o.d. or b.d.  Dose titration: 5 mg daily at weekly intervals until an optimum response is achieved. Usual effective dose of 20-25 mg daily (two divided doses).  Max dose: Dose not specified.^a^ |
| Dexamfetamine IR | **Aspen Dexamfetamine**  Approved age: ≥3 years  Initial dose: 2.5 mg daily.  Dose titration: 2.5 mg daily at weekly intervals.  Max dose: 40 mg daily in two divided doses. | **Dexedrine®**  Approved age: ≥6 years  Initial dose: 5 mg o.d. or b.d.  Dose titration: 5 mg daily at weekly intervals.  Max dose: Only in rare cases will it be necessary to exceed a total of 40 mg per day. | **Dexamfetamine**  Approved age: ≥3 years  Initial dose: *3-5 years:* 2.5 mg daily, *≥6 years:* 5 mg o.d. or b.d.  Dose titration: *3-5 years:* 2.5 mg daily at weekly intervals,  *≥6 years:* 5 mg daily at weekly intervals.  Max dose: Dose not specified.^a^ | **Amfexa®, dexamfetamine solution**  Approved age: ≥6 years  Initial dose: *≥6 years:* 5 mg o.d. or b.d.  Dose titration: 5 mg daily at weekly intervals.  Max dose: Usually 20 mg daily, although 40 mg daily may be needed in rare cases. | **Zenzedi®, Procentra®**  Approved age: ≥3 years  Initial dose: *3-5 years:* 2.5 mg daily, *≥6 years:* 5 mg o.d. or b.d.  Dose titration: *3-5 years:* 2.5 mg daily at weekly intervals,  *≥6 years:* 5 mg daily at weekly intervals.  Max dose: *3-5 years:* Dose not specified.^a^  *≥6 years:* Only in rare cases will it be necessary to exceed a total of 40 mg per day. |
| Dexamfetamine XR (sustained release formulation) |  | **Dexedrine® Spansule®**  Approved age: ≥6 years  Initial dose: 5 mg o.d.  Dose titration: 5 mg daily at weekly intervals.  Max dose: Only in rare cases will it be necessary to exceed a total of 40 mg per day. |  |  | **Dexedrine® Spansule®**  Approved age: ≥6 years  Initial dose: 5 mg o.d.  Dose titration: 5 mg daily at weekly intervals.  Max dose: Only in rare cases will it be necessary to exceed a total of 40 mg per day. |
| Dexamfetamine transdermal patch |  |  |  |  | **Xelstrym™**  Approved age: ≥6 years  Initial dose: 4.5mg/9 hours  Dose titration: 4.5mg weekly  Max dose: 18mg/9 hours |
| Lisdexamfetamine IR | **Vyvanse®**  Approved age: ≥6 years  Initial dose: 30 mg mane (or 20 mg if deemed appropriate)  Dose titration: 20 mg daily at intervals no more frequently than weekly  Max dose: 70 mg daily | **Vyvanse®**  Approved age: ≥6 years  Initial dose: 30 mg mane (or 20 mg if deemed appropriate)  Dose titration: 10 or 20 mg daily at approximately weekly intervals.  Max dose: 60 mg daily | **Vyvanse®**  Approved age: ≥6 years  Initial dose: 30 mg mane  Dose titration: 20 mg daily at intervals no more frequently than weekly  Max dose: 70 mg daily | **Elvanse®**  Approved age: ≥6 years  Initial dose: 30 mg mane (or 20 mg if deemed appropriate)  Dose titration: 10 or 20 mg daily at approximately weekly intervals.  Max dose: 70 mg daily | **Vyvanse®**  Approved age: ≥6 years  Initial dose: 30 mg mane  Dose titration: 10 or 20 mg daily at approximately weekly intervals.  Max dose: 70 mg daily |
| Methylphenidate transdermal patch |  |  |  |  | **Daytrana®**  Approved age: ≥6 years  Initial dose: 10 mg (applied to hip area 2 hrs before effect is needed, and should be removed 9 hrs after application, or earlier if needed).  Dose titration: Week 1 (10 mg) Week 2 (15 mg), Week 3 (20 mg) and Week 4 (30 mg). Dosage should be titrated to effect.  Max dose: the 30 mg/9 hour patches are the highest strength available. |
| Methylphenidate IR | **Ritalin®**  Approved age: ≥6 years  Initial dose: 5 mg o.d. or b.d.  Dose titration: 5–10 mg daily at weekly intervals  Max dose: Dose not specified^a^ | **Pms-Methylphenidate**  Approved age: ≥6 years  Initial dose: 5 mg t.d.s  Dose titration: 5–10 mg daily at weekly intervals  Max dose: 60 mg daily | **Ritalin®**  Approved age: ≥6 years  Initial dose: 5 mg o.d. or b.d.  Dose titration: 5–10 mg daily at weekly intervals  Max dose: 60 mg daily | **Medikinet®**  Approved age: ≥6 years  Initial dose: 5 mg o.d. or b.d.  Dose titration: 5–10 mg daily at weekly intervals  Max dose: 60 mg daily | **Ritalin®, Methylin®, Methylphenidate chewable tablets (generic brand)**  Approved age: ≥6 years  Initial dose: 5 mg b.d.  Dose titration: 5–10 mg daily at weekly intervals  Max dose: 60 mg daily |
| Methylphenidate XR (sustained release formulation) |  | **APO-methylphenidate SR**  Approved age: ≥6 years  Initial dose: May use corresponding doses in place of conventional methylphenidate tabs, when a prolonged effect is required (8-hour duration of action).  Dose titration: See initial dose.  Max dose: 60 mg daily | **Rubifen® SR**  Approved age: ≥6 years  Initial dose: May use corresponding doses in place of conventional methylphenidate tabs, when a prolonged effect is required (8-hour duration of action).  Dose titration: See initial dose.  Max dose: 60 mg daily |  | **Ritalin-SR®**  Approved age: ≥6 years  Initial dose: May use corresponding doses in place of conventional methylphenidate tabs, when a prolonged effect is required (8-hour duration of action).  Dose titration: See initial dose.  Max dose: 60 mg daily |
| Methylphenidate XR (outer DR coating/inner XR coating)^d^ |  |  |  |  | **Jornay PM®**  Approved age: ≥6 years  Initial dose: 20 mg in the evening. Adjust timing of administration between 6:30pm-9:30pm to optimise tolerability and efficacy the next morning and throughout the day.  Dose titration: 20 mg daily at weekly intervals  Max dose: 100 mg daily |
| Methylphenidate XR (20% IR, 80% XR components) |  |  |  |  | **Quillivant XR®**  Approved age: ≥6 years  Initial dose: 20 mg mane  Dose titration: 10-20 mg daily at weekly intervals  Max dose: 60 mg daily |
| Methylphenidate XR (multilayer beads: 20% IR layer, 80% XR layer) |  | **Foquest®**  Approved age: ≥6 years  Initial dose: *new to methylphenidate:* 25 mg mane. *Switching from another methylphenidate product:* give the next lower strength of Foquest, based on current total daily dose of methylphenidate  Dose titration: intervals of no less than 5 days.  Max dose: 70 mg daily |  |  | **Adhansia XR®**  Approved age: ≥6 years  Initial dose: 25 mg mane.  Dose titration: 10-15 mg daily, at intervals of no less than 5 days.  Max dose: 85 mg daily |
| Methylphenidate XR (22% IR, 78% XR components) | **Concerta®**  Approved age: ≥6 years  Initial dose: *new to methylphenidate:* 18 mg mane. *Switching from IR methylphenidate:* 18 mg (previously 5 mg t.d.s IR) or 36 mg (previously 10 mg t.d.s IR) or 54 mg (previously 15 mg t.d.s IR) mane.  Dose titration: 9 mg daily (from 18-36 mg) at weekly intervals, and then 18 mg daily at weekly intervals.  Max dose: *≥6 years:* 54 mg mane. | **Concerta®**  Approved age: ≥6 years  Initial dose: *new to methylphenidate:* 18 mg mane. *switching from IR methylphenidate*: 18 mg (previously 5 mg b.d.-t.d.s IR or 20mg SR d) or 36 mg (previously 10 mg b.d.-t.d.s IR or 40mg SR d) or 54 mg (previously 15 mg b.d.-t.d.s IR or 60mg SR d).  Dose titration: Adjust at weekly intervals.^e^  Max dose: *≥6 years:* 54 mg mane for patients new to methylphenidate. Dose not specified^a^ for patients switching from a current methylphenidate regimen. | **Concerta®**  Approved age: ≥6 years  Initial dose: *new to methylphenidate:* 18 mg mane. *Switching from IR methylphenidate:* 18 mg (previously 5 mg t.d.s IR) or 36 mg (previously 10 mg t.d.s IR) or 54 mg (previously 15 mg t.d.s IR) or 72 mg (previously 20 mg t.d.s IR) mane.  Dose titration: 9 mg daily (from 18-36 mg) at weekly intervals, and then 18 mg daily at weekly intervals.  Max dose: *6-12 years*: 54 mg mane, *≥13 years*: 72 mg mane. | **Concerta® XL**  Approved age: ≥6 years  Initial dose: *new to methylphenidate:* 18 mg mane. *Switching from IR methylphenidate:* 18 mg (previously 5 mg t.d.s IR) or 36 mg (previously 10 mg t.d.s IR) or 54 mg (previously 15 mg t.d.s IR) mane.  Dose titration: 18 mg daily at weekly intervals.  Max dose: *≥6 years:* 54 mg mane. | **Concerta®**  Approved age: ≥6 years  Initial dose: *new to methylphenidate:* 18 mg mane. *switching from IR methylphenidate*: 18 mg (previously 5 mg b.d.-t.d.s IR) or 36 mg (previously 10 mg b.d.-t.d.s IR) or 54 mg (previously 15 mg b.d.-t.d.s IR) or 72 mg (previously 20 mg b.d.-t.d.s IR) mane.  Dose titration: 18 mg daily at weekly intervals.  Max dose: *6-12 years*: 54 mg mane, *≥13 years*: 72 mg mane, not to exceed 2 mg/kg/day. |
| Methylphenidate XR (25% IR, 75% XR components) |  |  |  |  | **Cotempla XR-ODT™**  Approved age: ≥6 years  Initial dose: 17.3 mg mane  Dose titration: 8.6-17.3 mg daily at weekly intervals  Max dose: 51.8 mg daily |
| Methylphenidate XR (30% IR, 70% XR components) |  |  |  |  | **Quillichew ER®^f^**  Approved age: ≥6 years  Initial dose: 20 mg mane  Dose titration: 10, 15 or 20 mg daily at weekly intervals  Max dose: 60 mg daily |
| Methylphenidate XR (30% IR, 70% XR bead components) |  |  |  | **Equasym XL®**  Approved age: ≥6 years  Initial dose: Usually start treatment with IR formulation and then continue with XR formulation as the same total daily dose. If impracticable, may start with 10 mg mane in place of IR formulation (5 mg b.d.).  Dose titration: Dose titration is normally achieved using IR formulation.  Max dose: 60 mg daily | **Methylphenidate HCl CD XR capsule (TEVA generic brand)^f^**  Approved age: 6-15 years  Initial dose: 20 mg mane.  Dose titration: 10-20 mg daily at weekly intervals  Max dose: 60 mg daily |
| Methylphenidate XR (40% IR, 60% XR) |  | **Biphentin®**  Approved age: ≥6 years  Initial dose: *new to methylphenidate:* 10-20 mg mane. *Switching from IR methylphenidate:* convert to next lower strength of Biphentin, based on current methylphenidate total daily dose.  Dose titration: 10 mg daily at weekly intervals  Max dose: Max 60 mg daily. Some children may need higher doses (max 1 mg/kg/day). |  |  | **Aptensio XR®**  Approved age: ≥6 years  Initial dose: 10 mg mane.  Dose titration: 10 mg daily at weekly intervals  Max dose: 60 mg daily |
| Methylphenidate XR (50% IR, 50% XR)^g^ | **Ritalin LA®**  Approved age: ≥6 years  Initial dose: *new to methylphenidate:* 20 mg mane (or 10 mg if deemed appropriate).  *switching from IR methylphenidate:* continue with the same total daily dose, as a once-daily dosage.  Dose titration: 10 mg daily at weekly intervals  Max dose: 60 mg daily |  | **Ritalin LA®**  Approved age: ≥6 years  Initial dose: *new to methylphenidate:* 20 mg mane (or 10 mg if deemed appropriate).  *switching from IR or SR methylphenidate:* continue with the same total daily dose, as a once-daily dosage.  Dose titration: 10 mg daily at weekly intervals  Max dose: 60 mg daily | **Medikinet® XL^g^**  Approved age: ≥6 years  Initial dose: Usually start treatment with IR-F and then continue with Medikinet XL as the same total daily dose. If impracticable, may start with 10 mg mane in place of IR-F (5 mg b.d.).  Dose titration: Dose titration is normally achieved using IR-F.  Max dose: 60 mg daily | **Ritalin LA®**  Approved age: ≥6 years  Initial dose: *new to methylphenidate:* 20 mg mane (or 10 mg if deemed appropriate).  *switching from IR or SR methylphenidate:* continue with the same total daily dose, as a once-daily dosage.  Dose titration: 10 mg daily at weekly intervals  Max dose: 60 mg daily |
| Dexmethylphenidate IR |  |  |  |  | **Focalin®**  Approved age: ≥6 years  Initial dose: *new to methylphenidate:* 2.5 mg b.d. (4 hours apart).  *Currently using methylphenidate:* initiate Focalin therapy with half the current total daily dose of racemic methylphenidate.  Dose titration: 2.5-5 mg daily at weekly intervals.  Max dose: 20 mg daily (10 mg b.d.) |
| Dexmethylphenidate XR (50% IR, 50% DR) |  |  |  |  | **Focalin XR®**  Approved age: ≥6 years  Initial dose: *new to methylphenidate:* 5 mg mane.  *Currently using methylphenidate:* initiate Focalin XR therapy with half the current total daily dose of racemic methylphenidate.  *Currently using dexmethylphenidate IR:* Give total daily dose of IR tablets as o.d. dose of XR caps.  Dose titration: 5 mg daily at weekly intervals.  Max dose: 30 mg daily. |
| dexmethylphenidate and serdexmethylphenidate IR |  |  |  |  | **Azstarys™**  Approved age: ≥6 years  Initial dose: 39.2mg serdexmethylphenidate/7.8mg dexmethylphenidate daily  Dose titration: Increase dose to 52.3mg serdexmethylphenidate/10.4mg demethylphenidate o.d. after 1 week. For patients 6-12 years, may otherwise decrease to 26.1mg serdexmethylphenidate/5.2mg dexmethylphenidate if needed.  Max dose: 52.3mg serdexmethylphenidate/10.4mg demethylphenidate o.d. |
| Atomoxetine IR | **Strattera®**  Approved age: ≥6 years  Initial dose: *≤70 kg bodyweight:* 0.5 mg/kg daily, *>70 kg bodyweight:* 40 mg daily. Give daily doses as mane or b.d.; morning and late afternoon or early evening.  Dose titration: After a minimum of 3 days, increase to a target dose of approximately 1.2 mg/kg/day (≤70 kg bodyweight) or 80 mg daily (>70 kg bodyweight). After 2-4 weeks, may increase the above to respective max dose.  Max dose: *≤70 kg bodyweight*: 1.4 mg/kg or 100 mg daily, whichever is less. *>70 kg bodyweight:* 100 mg daily. | **Strattera®**  Approved age: ≥6 years  Initial dose: *≤70 kg bodyweight:* 0.5 mg/kg daily, *>70 kg bodyweight:* 40 mg daily. Give daily doses as mane or b.d.; morning and late afternoon or early evening.  Dose titration: After 7-14 days, increase to 0.8 mg/kg/day (*≤70 kg bodyweight)* or 60 mg daily (*>70 kg bodyweight).* After another 7-14 days, increase to approximately 1.2 mg/kg/day (*≤70 kg bodyweight)* or 80 mg daily (*>70 kg bodyweight).* After a minimum of 30 days, reassess and adjust maintenance dose (≤70 kg bodyweight) or for patients >70 kg, may increase to max dose after 2-4 weeks if necessary.  Max dose: *≤70 kg bodyweight*: 1.4 mg/kg or 100 mg daily, whichever is less. *>70 kg bodyweight:* 100 mg daily. | **Strattera®**  Approved age: ≥6 years  Initial dose: *≤70 kg bodyweight:* 0.5 mg/kg daily, *>70 kg bodyweight:* 40 mg daily. Give daily doses as mane or b.d.; morning and late afternoon or early evening.  Dose titration: After a minimum of 3 days, increase to a target dose of approximately 1.2 mg/kg/day (≤70 kg bodyweight) or 80 mg daily (>70 kg bodyweight). After 2-4 weeks, may increase the above to respective max dose.  Max dose: *≤70 kg bodyweight*: 1.4 mg/kg or 100 mg daily, whichever is less. *>70 kg bodyweight:* 100 mg daily. | **Strattera®**  Approved age: ≥6 years  Initial dose: *≤70 kg bodyweight:* 0.5 mg/kg daily, *>70 kg bodyweight:* 40 mg daily. Give daily doses as mane or b.d.; morning and late afternoon or early evening.  Dose titration: After a minimum of 7 days, increase to a maintenance dose of approximately 1.2 mg/kg/day (*≤70 kg bodyweight) or 80 mg daily (>70 kg bodyweight).*  Max dose: *≤70 kg bodyweight:* Dose not specified.^a^ *For >70 kg bodyweight:* 100 mg daily. | **Strattera®**  Approved age: ≥6 years  Initial dose: *≤70 kg bodyweight:* 0.5 mg/kg daily, *>70 kg bodyweight:* 40 mg daily. Give daily doses as mane or b.d.; morning and late afternoon or early evening.  Dose titration: After a minimum of 3 days, increase to a target dose of approximately 1.2 mg/kg/day (≤70 kg bodyweight) or 80 mg daily (>70 kg bodyweight). After 2-4 weeks, may increase to max dose (>70 kg bodyweight).  Max dose: *≤70 kg bodyweight*: 1.4 mg/kg or 100 mg daily, whichever is less. *>70 kg bodyweight:* 100 mg daily. |
| Clonidine XR (extended release formulation) |  |  |  |  | **Kapvay®**  Approved age: ≥6 years  Initial dose: 0.1 mg daily (bedtime)  Dose titration: Adjust by 0.1 mg daily at weekly intervals until desired response. Take total daily doses as follows; 0.2 mg daily (as 0.1 mg mane and bedtime), 0.3 mg daily (as 0.1 mg mane and 0.2 mg bedtime) and 0.4 mg daily (as 0.2 mg mane and bedtime).  Max dose: 0.4 mg daily. |
| Guanfacine XR (extended release formulation) | **Intuniv®**  Approved age: ≥6 years  Initial dose: 1 mg daily.  Dose titration: Adjust by 1 mg daily at weekly intervals to recommended maintenance dose of 0.05-0.12 mg/kg/day (total daily dose of 1-7 mg).  Max dose: *6-12 years (>25 kg):* 4 mg daily, *≥13 years:* 7 mg daily. Max 4 mg when used as an adjunct to psychostimulants. | **Intuniv XR®**  Approved age: ≥6 years  Initial dose: 1 mg daily.  Dose titration: Adjust by 1 mg daily at weekly intervals to recommended maintenance dose of 0.05-0.12 mg/kg/day (total daily dose of 1-7 mg).  Max dose: *6-12 years (>25 kg):* 4 mg daily, *≥13 years:* 7 mg daily. Max 4 mg when used as an adjunct to psychostimulants. |  | **Intuniv®**  Approved age: ≥6 years  Initial dose: 1 mg daily.  Dose titration: Adjust by 1 mg daily at weekly intervals to recommended maintenance dose of 0.05-0.12 mg/kg/day (total daily dose of 1-7 mg).  Max dose: *6-12 years (>25 kg):* 4 mg daily. *≥13 years (minimum 34 kg):* maximum 4 mg daily *(34-41.4 kg) or* 5 mg daily (*41.5-49.4 kg) or* 6 mg daily (*49.5-58.4 kg) or 7* mg daily (*≥58.5 kg)*. | **Intuniv®**  Approved age: ≥6 years  Initial dose: 1 mg daily.  Dose titration: Adjust by 1 mg daily at weekly intervals to recommended maintenance dose of 0.05-0.12 mg/kg/day (total daily dose of 1-7 mg).  Max dose: *6-12 years (>25 kg):* 4 mg daily, *≥13 years:* 7 mg daily. Max 4 mg when used as an adjunct to psychostimulants. |

Formulation abbreviations: immediate-release (IR), extended-release (XR), orally-disintegrating tablet (ODT), extended-release tablet (XR-T), extended-release capsule (XR-C), extended-release suspension (XR-S), extended-release orally-disintegrating tablet (XR-ODT).

Other abbreviations: once daily (o.d.), once daily in the morning (mane), twice daily (b.d.), three times daily (t.d.s.).

grey fill: not marketed in the respective country.

^a^Dose not specified in corresponding drug labeling.

^b^Dyanavel XR**®** (solution and tablets) contain both IR and XR components of amfetamine, although the ratio of IR to XR is unknown. Reference: Childress AC, Kollins SH, Foehl HC, et al. Randomized, Double-Blind, Placebo-Controlled, Flexible-Dose Titration Study of Methylphenidate Hydrochloride Extended-Release Capsules (Aptensio XR) in Preschool Children with Attention-Deficit/Hyperactivity Disorder. *J Child Adolesc Psychopharmacol*. Mar 2020;30(2):58-68. doi:10.1089/cap.2019.0085

^c^*“…They contain three types of drug-releasing beads, an immediate release and two different types of delayed release (DR) beads. The first DR bead releases amphetamine at pH 5.5 and the other DR bead releases amphetamine at pH 7.0.”* Reference: Mydayis (2022). Prescribing Information. Takeda Pharmaceuticals America, Inc. Available from <https://dailymed.nlm.nih.gov/dailymed/index.cfm> [Accessed March 8, 2023].

^d^ *“…JORNAY PM extended-release capsules contain beads with two functional film coatings (outer delayed-release and inner extended-release) surrounding a drug core coated with methylphenidate hydrochloride. The outer, delayed-release coating delays the initial release of methylphenidate while the inner extended-release coating controls the release throughout the day…”, “…The initial absorption of methylphenidate into plasma is delayed such that no more than 5% of total drug is available within the first 10 hours after dosing. After the lag period, the absorption of methylphenidate occurs in a single peak with a median T 14.0 hours, followed by a gradual decline throughout the rest of the day…”* Reference: Jornay PM extended-release (2022). Prescribing Information. Ironshore Pharmaceuticals Inc. Available from <https://dailymed.nlm.nih.gov/dailymed/index.cfm> [Accessed March 8, 2023].

^e^Although complete dosage adjustment guidelines are not provided in the Canadian Product Monograph, Concerta is available in 18 mg, 27 mg, 36 mg, 54 mg and 72 mg strengths, which would be expected to guide dosage adjustments. Reference: Concerta (2022). Product Monograph. Janssen Inc. Available from <https://health-products.canada.ca/dpd-bdpp/> [Accessed March 8, 2023].

^f^ Quillichew ER**®** (30% IR, 70% XR components) differs from methylphenidate CD TEVA brand (30% IR, 70% XR beads components) due to the pharmacokinetic profile and extended-release technology, so they were treated as different drug formulations.

^g^ Medikinet**®** LA shown to be bioequivalent to Ritalin LA under fasting conditions, but NOT in the fed state. Reference: Haessler F, Tracik F, Dietrich H, Stammer H, Klatt J. A pharmacokinetic study of two modified-release methylphenidate formulations under different food conditions in healthy volunteers. *Int J Clin Pharmacol Ther*. Sep 2008;46(9):466-76. doi:10.5414/cpp46466
